# Optimal Eye-Gaze Fixation Position for Face-Related Neural Responses

**DOI:** 10.1371/journal.pone.0060128

**Published:** 2013-06-06

**Authors:** Younes Zerouali, Jean-Marc Lina, Boutheina Jemel

**Affiliations:** 1 Ecole de Technologie Supérieure, Montreal, Canada; 2 Hôpital Riviere des Prairies, Montreal, Canada; 3 Centre de Recherches Mathématiques, Université de Montréal, Montreal, Canada; 4 School of Speech Language Pathology, faculty of medecine, Université de Montréal, Montreal, Canada; Cuban Neuroscience Center, Cuba

## Abstract

It is generally agreed that some features of a face, namely the eyes, are more salient than others as indexed by behavioral diagnosticity, gaze-fixation patterns and evoked-neural responses. However, because previous studies used unnatural stimuli, there is no evidence so far that the early encoding of a whole face in the human brain is based on the eyes or other facial features. To address this issue, scalp electroencephalogram (EEG) and eye gaze-fixations were recorded simultaneously in a gaze-contingent paradigm while observers viewed faces. We found that the N170 indexing the earliest face-sensitive response in the human brain was the largest when the fixation position is located around the nasion. Interestingly, for inverted faces, this optimal fixation position was more variable, but mainly clustered in the upper part of the visual field (around the mouth). These observations extend the findings of recent behavioral studies, suggesting that the early encoding of a face, as indexed by the N170, is not driven by the eyes *per se*, but rather arises from a general perceptual setting (upper-visual field advantage) coupled with the alignment of a face stimulus to a stored face template.

## Introduction

During real-world scene viewing, visual information is sampled via successive changes of gaze-fixation positions, resulting in continuously changing projections of our visual field on the retina. Given the heterogeneity of retinal photoreceptors' distribution, the position of gaze fixation within a visual display, faces in our case, determines the quality of encoding of each face region. Understanding the optimal encoding of a face stimulus with respect to retinal sampling, i.e. finding the optimal gaze fixation position, constitutes a critical step toward understanding the driving principles of face-related neural responses.

It is generally agreed that the earliest neural representation of a face is detected in the EEG about 170 milliseconds after stimulus presentation, and manifests as a negative ERP component (N170) over occipito-temporal electrode sites [1]. The N170 is thought to reflect the earliest stages of face processing. Moreover, the amplitude of the N170 was linked to the strength of face representations in the brain. Indeed, N170 amplitude is modulated by spatial attentional load during the encoding of face stimuli [2]. In addition, the N170 amplitude decreases gradually with the amount of white noise [3] and phase-spectrum noise [4] in the stimulus. It is thus reasonable to assume that gaze fixation position within a face would modulate N170 amplitude by affecting the quality of encoding of each face region. More importantly, the optimal face encoding can be revealed by measuring the N170 amplitude elicited by different gaze fixations.

**Figure 1:**
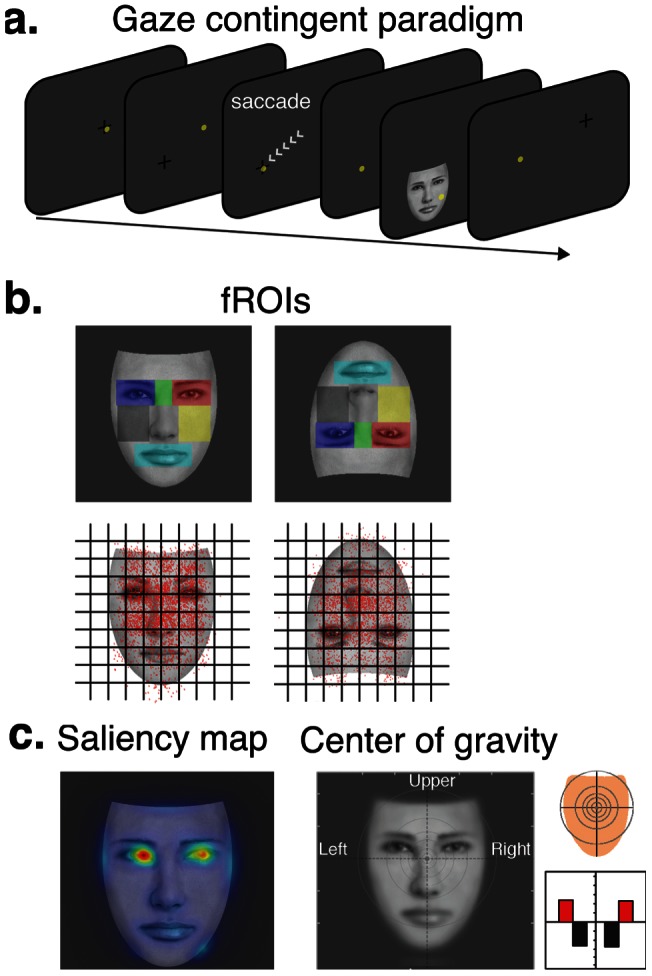
Illustration of the experimental protocol. (**a**) Schematic view of a trial progression during the eye-gaze contingent paradigm used in this study. On the beginning of each trial, a fixation-cross is presented randomly on the screen, and is replaced by a face image as soon as the eye-tracker detects that the observer's line of sight lays on the fixation-cross. The yellow circles represent the observer's gaze position on the screen. Eye-fixation landing position on the seven predefined fROI (**b**) is controlled by the presentation of a fixation-cross located randomly in one of nine invisible quadrants on the screen, that were spatially mapped with the location of the fROIs (top). Actual gaze positions compiled over all subjects and trials for each face orientation (bottom). Gaze position for each trial is depicted with a black dot. (**c**) Saliency map and spatial resolution of face images and visual field map coverage when the centre of gravity of the face is fixated.

Several lines of evidence suggest that face processing is optimized when gaze fixation focuses on the eyes. Indeed, the eyes are the most salient features of the face based on its energy density spectrum [5], [6] (see fig. 1c). When presented alone, the eyes evoke a N170 response with higher amplitude than any other face parts, and even higher than to whole faces [7]. Meanwhile, face pictures evoke similar N170 amplitudes whether the eyes are present or removed [8], [9]. To reconcile these findings, some authors suggested that the N170 results from an interplay between a holistic face-processing system and specialized eye-processing system. According to this interpretation, the N170 reflects the holistic processing of faces; however, when holistic information is not available, either using isolated faces parts or inverted faces, the eye-processor is brought to play [8]. However the existence of an eye-specialized brain structure remains speculative and was contradicted by recent findings from McPartland and colleagues [10], who found that gaze fixations between the eyes (i.e., the nasion) or on the mouth evoke N170 responses with similar amplitudes.

Another line of evidence suggests that face processing might rather be optimized by fixating the center of gravity of the face, which is located around the center of the face, slightly below the eyes [11]–[13]. Interestingly, the center of gravity is thought to optimize the holistic face processing: in contrast to normal observers, a brain-damaged patient who does not process faces holistically does not fixate on this position but rather precisely on each specific feature, i.e., mouth, right and left eyeball [14]. Moreover, gaze fixations on inverted faces, which are not processed holistically [15], tend to depart from the center of gravity with increased fixations on or towards the mouth of the face [16],[17]. These findings are however drawn from behavioral studies and do not allow dissociation between early face processing and task performance.

Here, in order to find the gaze-fixation position that optimizes the earliest face representation in the human brain, we reasoned that one needs to stimulate with whole faces, and yet take the challenge of determining which area of the face drives the strongest N170 response amplitude. MacPartland and colleagues [22] approached this question by studying brain responses to cued fixations within faces; in turn, we propose to study the same brain responses to the truly fixated locations within faces (the implications of this methodological divergence are exposed in the methods, section 4, and in the discussion, section 3). To do so, we introduce a new methodological approach that uses simultaneous EEG and eye-tracking measures to precisely map single-trial N170 amplitude as a function of the viewers' eye-gaze position on a face display. In order to ensure the effects of point of fixation are specific to perceptual process, we also record the P100 ERP component which is a pre-categorical component, and is mainly sensitive to low-level features of the stimulus. We employ a modified version of the gaze-contingent paradigm, in which we probe the viewers' eye-landing positions with a fixation cross displayed at different locations on the screen (fig. 1a&b). By fixating the cross, the viewer triggers the presentation of a face image. The face stimulus is displayed for a short duration that is sufficient for us to track one gaze-fixation position (whose average time is approximately 200–300 ms) at each trial. It is worth noting that by allowing only one fixation to occur, we replicate the conditions under which the N170 response is recorded in most face ERP studies. In addition, we tested the eye-specialized processor hypothesis by assessing the behavior of the N170 generators with respect to fixated face regions.

Under this paradigm, we asked whether the quality of face representations in the brain depends on a particular face region, notably the eyes (see fig. 1c) or on the spatial distribution of face features as predicted by the center of gravity. Because they offer the best control for upright face stimulation, we also presented inverted faces in this study. By using large face displays, we enforced large changes in retinal projection as a function of fixation point. We hypothesized that optimal viewing point for face processing would fall on the center of gravity on upright stimuli, as suggested by previous studies [11]–[13]. However, for inverted faces, we predicted that the largest N170 would be observed for fixations located towards the mouth. Finally, we hypothesized that the existence of a specialized eye-processor would be revealed by reconstructing the N170 evoked by fixations at the eyes but not on other face regions.

## Materials and Methods

### Participants

Nineteen healthy young adults (11 female, mean age 23.4 years ±4.3) took part in the experiment. All observers were right handed, and had normal or corrected-to-normal vision and no history of neurological or psychiatric disorder. We excluded from the experiment participants under cortisone medication due to possible hypersensitivity to infrared light. All participants gave written informed consent, approved by the Ethics Board of Riviere-des-Prairies Hospital. Data from two participants were excluded from the analysis due to excessive artifacts over occipital electrodes.

### Stimuli

We constructed 100 grey scale front-view faces (half female) with no hair or barb, using Faces software (Faces ID, Kingwood, Texas). Each face was scaled to match approximately the eyes, nose, and mouth positions across the whole face set. Face stimuli were presented in an upright or inverted orientation on a 21 inch CRT monitor (ViewSonic G225f; spatial resolution 1024×768, refresh rate 80 Hz). Observers were seated 62 cm away from the screen with their head stabilized with a chin-rest. At this distance, face stimuli occupied 17°×23°. The predefined face regions of interest (fROIs) that were targeted in our study encompassed seven facial features; the left and right eyes including eyebrows (6.1°×4.8° each), the nasion (6.1°×3.9°), the left and right jaws (5.1°×6.6° each), the nose (7.3°×6.6°), and the mouth (7.9°×4.4°).

### Eye-tracking apparatus

A monocular eye-tracking camera with infrared illuminator (Eyelink II 1000, SR Research Ltd., Canada) was positioned 50 cm away from the observer and sampled eye position every millisecond. The standard nine-point EyeLink II calibration procedure was administered at the beginning of each experimental block, and was repeated whenever the drift-correction error was larger than 1° of visual angle. Observers were required to maintain fixation centered around the fixation cross(2° of visual angle), and to move their gaze towards its location whenever the fixation cross disappears and reappears in another position on the screen.

### Procedure

In free-viewing conditions, it was consistently found that the preferred landing positions for eye-fixations are clustered around the upper part of the face [11], [12]. In order to avoid such bias, we developed a version of the gaze-contigent paradigm aimed at sampling equally the seven predefined fROIs across trials (see section). Each trial was aimed at sampling a single fROI using a fixation cross that appeared before the face (fig. 1a). A blank screen (100 ms) separated the presentation of the cross (200 ms) and the presentation of the face (200 ms). In order to ensure subjects fixate the cued fROI, the face was only displayed if the cross was fixated continuously for 200 ms and the subjects were asked to keep fixating at that location during the blank screen. However, we noted that subjects made saccades during the blank screen on some trials, so the cued fROI differed from the **truly** fixated fROI. All the trials were thus labelled offline with respect to the **truly** fixated fROI using gaze fixation position within face displays (data on the truly fixated regions is provided in fig. 1b). It is important to note that we allowed only one fixation per trial; if the first fixation on the face display was shorter than 200 ms, the trial was rejected. In addition, the order in which fROIs were cued and the position of the face with respect to the center of the screen were completely unpredictable to the subjects. All participants completed four blocks of 230 trials each, where each fROI from upright and inverted faces were cued on approximately 66 trials. All blocks began with a camera calibration routine in order to ensure eye-tracking stability and were followed by a short break.

### Electrophysiological recordings

Electroencephalogram (EEG) signals were continuously recorded (DC-100 Hz band pass, 1024 Hz sampling rate) using 58 Ag/AgCl electrodes mounted in an Easy-cap (Brain Products, GmbH) according to the extended 10–20 system. Electrooculogram (EOG) activity was recorded with four additional electrodes located at the outer canthi of both eyes, and below and above the right eye. All electrodes were referenced to the left earlobe and their impedances were always kept below 5 kOhm. EEG signals were offline average-referenced and digitally filtered (0.3–30 Hz with a 24 dB/oct slope). Artifacts were rejected over a 200 ms sliding window using a 40
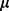
V standard deviation criterion on EOG electrodes and a 20
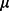
V standard deviation on all scalp electrodes. Blink artifacts were corrected by subtracting from the EEG the PCA-transformed EOG components for each electrode [18], weighted according to the VEOG propagation factors (computed via linear regression).

### ERP data analyses

EEG signals time-locked to the onset of upright and inverted face presentation were averaged separately for each fROI as defined from the eye-tracking data (true fixations, see section 0. ERPs included in all cases a pre-stimulus baseline of 200 ms and were 800 ms long. ERP amplitudes (N170 and P1) were analyzed using the multivariate repeated-measures approach (MANOVA) with the fROIs and electrode lateralization (contralateral vs. ipsilateral; relative to the cued location) as factors. Significant effects or interactions were further assessed using Bonferroni-corrected t-tests for pairwise comparisons.

### Heat maps

In order to clarify the effect of fixation positions on ERP amplitudes independently from fROIs, we mapped N170 and P100 peak amplitudes directly on a face stimulus. We compiled trials from all subjects and computed for each trial the (x,y) gaze-fixation position and the peak amplitude. This step only relies on the actual fixated location on each trial and not on the associated fROI. Compiled landing sites of all trials for the two conditions are displayed on fig. 1d. Trials were then grouped within equal-sized non-overlapping squares (100) that covered the whole stimulus and local averages were computed within each square. As the number of trials differs among squares, local averages were corrected with respect to the signal to noise ratio by dividing with the local standard deviation. We then used cubic interpolation to generate smooth ‘heat maps’ based on those 100 points (Matlab, The Mathworks Inc.). In order to assess statistical significance of the heat maps, we generate a distribution of random ERP amplitudes using surrogate data. We randomly permuted the ERP amplitude among (x,y) locations and computed surrogate heat maps (sMaps). In total, 200 SMaps were generated for each condition. Statistical significance of each point on the original maps was assessed by comparing amplitude at that point with the amplitude on the sMaps. 5% significance is reached if a point on the original map has amplitude greater than at least 180 sMaps.

### Source reconstruction

Using the MNI-Colin template brain, we computed the forward problem using a realistic 3-shell head model. In total, 15028 current dipoles, or sources 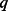, with constrained orientations were distributed on the cortical mantle. The gain matrix was generated using the OpenMEEG [19] software plugged into the Brainstorm analysis software [20]. Time courses of source intensities were reconstructed by solving the inverse problem with the Maximum Entropy on the Mean [21] (see Appendix S1) tools introduced in Brainstorm. We computed the grand average ERPs (gaERP) across all subjects for each face fixation position on the faces (upper, middle and lower regions). We localized the sources of each gaERP separately and we average the the time courses of the sources within the range of the N170 in order to get one cortical map of activity for each for each gaERP. These cortical maps were then normalized and averaged together. We defined active cortical regions by thresholding the averaged normalized map to 0.5. We then extracted the time course of the extracted cortical regions separately for each gaERP.

## Results

EEG data and eye-fixation positions were collected simultaneously from seventeen observers during an eye-gaze contingent paradigm (see fig. 1a). This paradigm allowed us to label single-trial ERPs with respect to the truly fixated fROIs. When each of the fROIs is foveated by the observer, vast changes in the retinal projection of the face stimulus were expected to occur both in terms of image spatial resolution and visual field coverage (see fig. S1). Artifact-free EEG epochs time-locked to the onset of upright and inverted face stimuli were averaged separately for each fROI. These ERP averages were then pooled to allow comparisons of ERP signals with respect to horizontal and vertical meridians of visual fields. Along with fROI labels, we also extracted the exact gaze fixation coordinates on each trial (fig. 1b), and used these coordinates to precisely map N170 and P100 peak amplitude on face displays.

### N170 amplitude and fixation position

**Figure 2:**
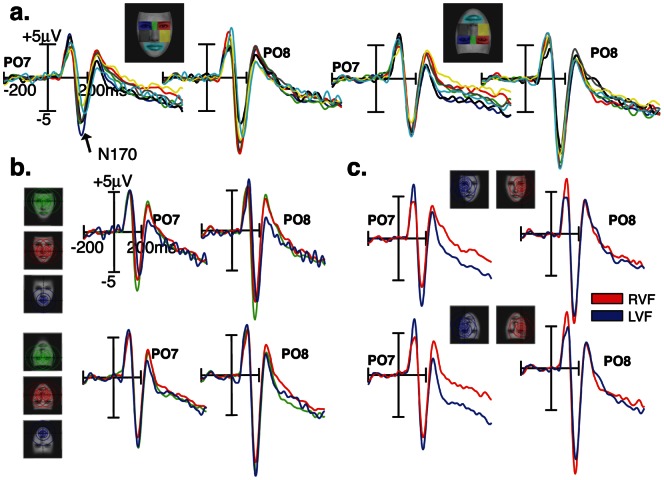
N170 ERP responses over PO7 and PO8 scalp sites are shown as a function of eye-gaze landing positions on face stimuli. (**a.**) N170 responses recorded at fixated face regions of interest (fROIs) in upright and inverted face images. (**b.**) N170 responses elicited by fixated face regions along the vertical meridian (upper in green, middle in red, and lower in blue) in upright and inverted face images. (**c.**) N170 responses elicited by fixated face regions along the horizontal meridian (left and right visual fields).

N170 amplitudes measured over lateral occipito-temporal (P7/8 and PO7/8) scalp sites (fig. 2a) varied with respect to fixated facial features (fROIs) for both upright [*F*(6,11)  = 4.42; *p* = 0.016] and inverted faces [*F*(6,11)  = 5.04; *p* = 0.01]. On visual inspection, it appears that in the upright face condition, the largest N170 responses were elicited when the upper face regions were fixated, namely the eyes and nasion, while the smallest responses were elicited by the lower face region (i.e., mouth). In the inverted face condition however, the N170 responses elicited by upper (mouth) and lower (eyes and nasion) face regions were no longer different, albeit there was a slight advantage for the upper part of the inverted face (mouth). This trend is confirmed by averaging the ERPs elicited by upper, middle and lower fROIs (fig. 2b).

**Figure 3:**
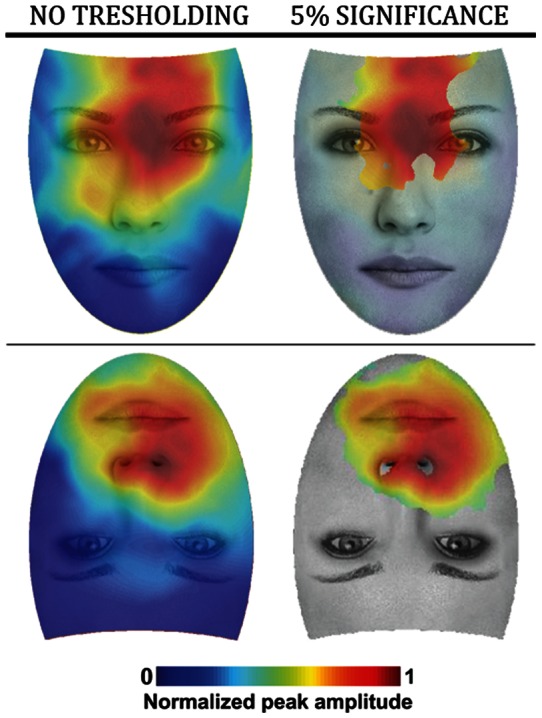
Heat maps for upright and inverted faces. Values of the heat maps indicate the relative strength of N170 response as a function of viewpoint (left). Bootstrap analysis was applied on the heat maps up to a p = 0.05 significance threshold (right).

In order to get a finer spatial picture of the electrical brain responses elicited by specific gaze-fixations positions, we mapped single trial EEG voltage amplitudes within the N170 time-range on the corresponding gaze positions on face displays. As detailed in the Methods section, we generated heat maps for upright and inverted faces, which highlight the fixated face regions that elicit strong N170 evoked responses. We refer to these regions as the “hot spots”. For the upright heat map, the hot spot for the N170 response is located on the nasion and spreads uniformly to adjacent face regions, and the cold spot is located on the mouth and lower regions (fig. 3a). It is worth noting that the hot spot does not cover the eyes' region *per se*. For the inverted heat map, the hot spot covers the tip of the nose and the mouth with a slight bias to the right visual hemifield, and the cold spot is located in the lower part of a displayed inverted face including the eyes' region (fig. 3b). Non parametric statistical thresholding to 5% level shows that only N170 response strengths covering the hot spot areas, both for upright and inverted faces, are significantly different from a random distribution of brain responses among facial regions (fig. 3c&d).

### P100 amplitude and fixation position

**Figure 4:**
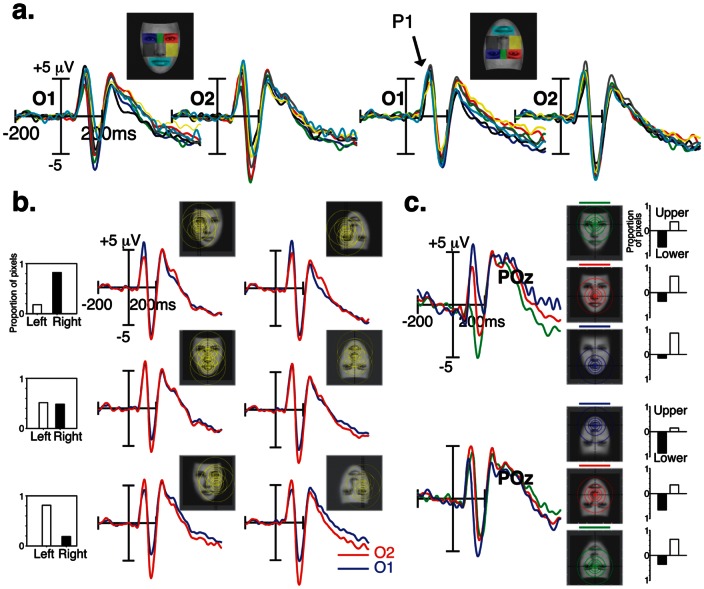
Sensitivity of the P100 waveform to fixation position. (**a**) P100 ERP responses to specific eye-gaze landing positions (fROI) are shown at occipital scalp sites (O1/O2) separately for upright and inverted faces. (**b**) P100 ERP waveforms recorded over O1 (in blue) and O2 (in red) electrodes are shown for fixated face regions along the horizontal meridian (left and right visual fields). (**c**) P100 ERP waveforms elicited by fixated face regions along the vertical meridian (upper in green, middle in red, and lower in blue).

In order to test whether this effect is specific to the N170 or reflects a general visual bias, we also investigated an earlier visual ERP component, the occipital P100 component (fig. 4a). For both face orientations, the MANOVA did not yield a significant effect of fROIs on the amplitude of P100, measured over O1/2 and PO7/8 [*F*(6,11) <1.08; *p*>0.4]. However, as illustrated in fig. 4a, the amount of information distributed across the left and right hemifields modulated P100 amplitude over the left and right hemispheres. This was reflected by a significant fROI by Hemisphere interaction for both face orientations [*F*(6,11) >3.3; *P* = 0.042]. When the fixated fROIs are located in the left field of the face stimulus, much of the facial information covered the right peripheral visual field, leading to larger P100 responses over the left than over the right occipital scalp sites [*P*<0.008]. The reverse pattern was found when the fixated fROIs are located in the right field of the face stimulus. No hemispheric difference was found when the eye-gaze fell on the middle of the face. fig. 2c shows the effect of eye-position along the horizontal meridian on P100 amplitude, measured over electrode POz [*F*(2,15)  = 13.98; *P*<0.0004]. For upright faces, P100 was larger to foveated mouth feature than the nose [*P* = 0.036], and to foveated nose feature than the nasion [*P* = 0.003]. This pattern was reversed when faces were presented upside-down [*F*(2,15)  = 4.38; *P*<0.035].

We generated heat maps of electrical brain activity at P100 time range as described for the N170 (fig. S2). We found that, for both stimulus orientations, upper face regions evoke positive ERP amplitude while lower regions evoke negative voltage ERPs. This effect is clearer for upright faces. However, after statistical thresholding, it appears that this ERP amplitude distribution is not significant for any face region (*p*>0.05).

### Asymmetrical ERP responses

The distribution of N170 amplitude over lateral scalp sites differed with respect to fixated face regions along the horizontal meridian [*F*(1,16) >12.69; *p*<0.003] (fig. 2c.). Although N170 amplitudes were generally larger over the right than over the left hemisphere [*F*(6,11) >20.33; *p*<0.0001], the right hemisphere advantage was more conspicuous when the fixated face regions fell in the right than in the left part of a face for either face orientations [*p*<0.006, Bonferroni corrected]. This hemisphere by fixated lateral hemifield interaction also shows N170 amplitude differences between fixated left and right face part over the left hemisphere [*p*<0.0001, Bonferroni corrected], and over the right hemisphere when faces are inverted [*p*<0.004, Bonferroni corrected].

Hemisphere by lateral hemifield interaction was also observed in P100 responses [*F*(6,11) >3.3; *p* = 0.042]. This interaction indicates that when the fixated fROIs falls in the left part of the face stimulus, much of the facial information occupies the right visual field, leading to larger P100 responses over the left than over the right occipital scalp sites [*p*<0.006, Bonferroni corrected] (fig. 4b). The reverse pattern was found when the right part of the face stimulus was fixated [*p*<0.015, Bonferroni corrected]. No hemispheric difference was found when the eye-gaze fell on the middle of the face [*p*>0.1]. The effect of eye-gaze position along the vertical meridian on P100 amplitude was found at midline parieto-occipital (POz) scalp site [*F*(6,11) >3.93; *p*<0.025].

### ERP Face inversion effect

**Figure 5:**
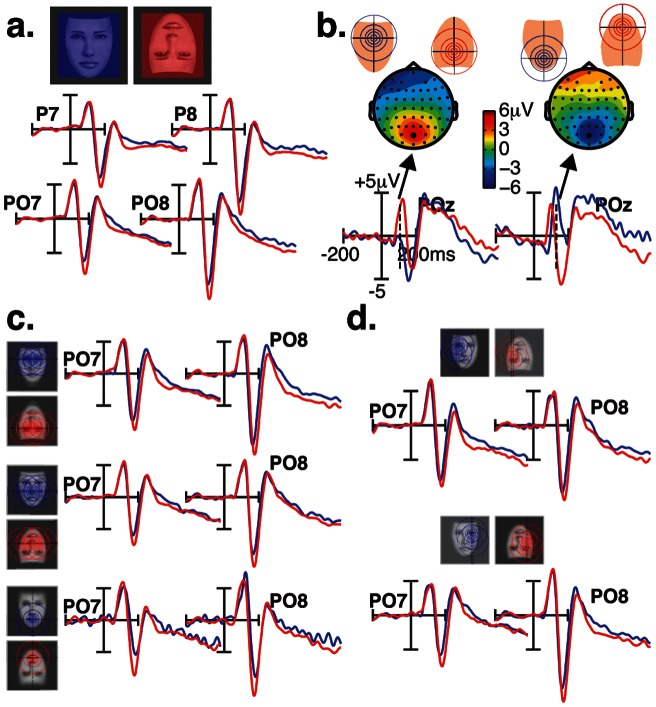
ERP face inversion effects. (**a.**). Pooled ERP responses elicited by all fROIs show the typical effect of face inversion on the N170. (**b.**). Scalp ERP difference (upright minus inverted faces) and ERP waveforms over POz are shown at P100 ERP latency range to foveated nasion (left) and mouth (right) features. (**c.**) N170 Face inversion effects for fixated face regions along the vertical meridian (upper, middle, and lower visual fields). (**d.**) N170 Face inversion effects for fixated face regions along the horizontal meridian (left and right visual fields).

Our findings are also relevant for the N170 face inversion effect, namely the increase of amplitude and latency that is found when faces are presented upside-down. We therefore compared the ERP responses to fixated face regions in the upright and inverted face conditions. At a macroscopic level (fig. 5a), pooled ERP responses elicited by all fROIs show the characteristic face inversion effect on the N170 peak. N170s were larger in amplitude and delayed in latency for inverted than for upright faces [*F*(1,16) >18.72; *p*<0.001]. No significant face inversion effect was found at P100 peak time-range recorded over lateral occipital sites [*F*(1,16) <2.24; *p*>0.1]. However, the effect of inversion on P100 is visible over midline parieto-occipital electrode POz, which took opposite expressions as a function of fixated fROIs along the vertical meridian (nasion, nose, mouth) [fROI by inversion interaction: *F*(1,16)  = 19.15; *p*<0.0001]. As shown in fig. 5b, P100 was larger when the nasion was fixated in an inverted than in an upright face [*p* = 0.0002, Bonferroni corrected], but was smaller in response to the mouth region when fixated in an inverted than in an upright face [*p* = 0.016, Bonferroni corrected]. No other effects on P100 were found. Interestingly, the magnitude of the N170 face inversion effect varied as a function of eye-gaze landing position, more specifically over left occipito-temporal electrodes. This was reflected by a significant interaction between Inversion, fROI, and Hemisphere [*F*(6,11)  = 4.48; *p* = 0.015]. As shown in fig. 5c, the N170 face inversion effect was larger when the fixated region was located in the lower part of the face (the mouth) than when located in the upper part of the face, over the left hemisphere [*p*<0.04, Bonferroni corrected].

### Neural sources activity

**Figure 6:**
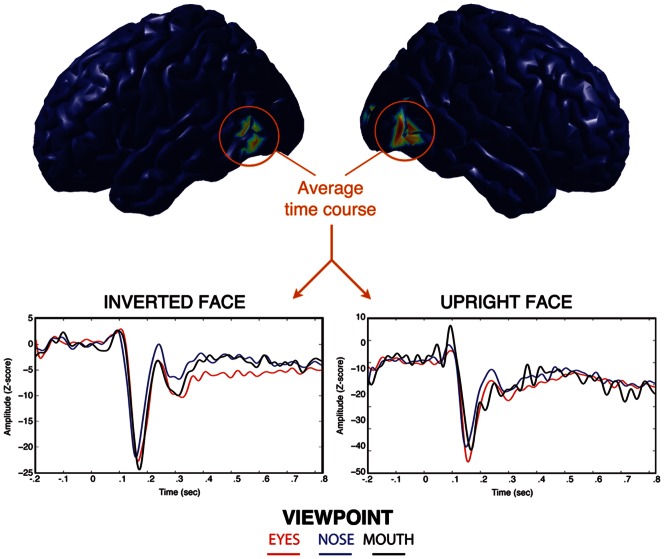
Reconstruction of the time course of sources active during the N170 evoked potential using the MEM technique. Bilateral sources found in the inferior temporal gyrus exhibit higher amplitude in response to fixations at the eyes of upright faces, and at the mouth of inverted faces.

Source reconstruction using the MEM [21] yielded two distinct cortical patches active during the time course of the scalp recorded N170 (fig. 6). This pattern of activity was consistent across fixated face regions and stimulus orientations. The two bilateral temporal patches lay along the caudal part of the inferior temporal gyrus (cITG). By reconstructing the time course of this region, we identified a peak at the same latency as the N170 and sharing similar amplitude patterns with respect to fROIs. Indeed, N170 peak amplitude at the sources level was evoked by fixations on the top of the face, irrespective of the planar orientation of stimuli.

## Discussion

In this contribution, we introduce a novel gaze-contingent paradigm in conjunction with electroencephalographic recordings to map the strength of electrical brain signal responses to various eye-fixation positions during the processing of natural face displays. In the following, we will discuss how the current findings relate to low-level properties of human visual field. We stress the fact that visual field quadrants are here understood as spatially dependent on the point of gaze fixation rather than on the center of the screen, and therefore they are not static. This distinction is important to bare in mind when interpreting neural responses to different fixation positions.

### Optimal fixation point for face processing

Firstly, ERP analyses and ERP heat maps showed that the N170 response to upright faces has maximal amplitude when gaze fixations fall on the nasion. This result is in line with findings from recent eye-tracking studies demonstrating that the preferred landing position of the viewer's first eye fixation is located at or slightly above the centre of the face [11]–[13]. In addition, N170 decreases gradually as fixation shifts downward, reflecting a graded modulation of the activity in face-responsive areas as a function of sampled visual information. This observation supports findings from a behavioral study showing that face encoding proceeds naturally in a downward chronological order, upper face features (i.e., the eyes) being processed first, followed by middle and bottom regions [22]. Our study thus extends this finding by suggesting that the nasion region is the physiologically most relevant starting point for face exploration.

Secondly, N170 response amplitude shows that optimal face representation of an inverted face is achieved by fixations at the mouth. ERP heat maps show that the N170 hot spot is between the mouth and the tip of the nose. This result is consistent with findings from behavioral studies that showed preferred fixations on or toward the mouth of inverted faces [16], [17]. Importantly, this result raises some doubt about the role of physical saliency of the eyes for encoding faces [5], [6]. One might argue that physical saliency of the eyes explains our maximal N170 amplitude at the nasion of upright faces. Indeed, this position could allow best encoding of both eyes simultaneously. However, this argument is not supported by our results with inverted faces, for which the optimal fixation position cannot explained by features' saliency.

### Where do the eyes stand?

There has been a wealth of research investigating the role of the eyes for encoding natural faces and yet the controversy still persists. On the one hand, it was shown that the eyes, when seen alone, evoke a higher N170 amplitude than a whole face [7], [8]. On the other hand, it was shown that a face evokes similar N170 amplitudes wether the eyes are present or not [9]. In order to reconcile these findings, it was proposed that the eyes trigger the activity of a specialized “eye-processor”, but only when the configural information of the face is disrupted (i.e. eyes shown alone or face inversion, [8]). However, our source reconstruction results contradict this hypothesis.

Our MEM source reconstruction (see section 0) yielded two bilateral sources in the inferior temporal gyrus (ITG). The activity of the ITG was recently correlated with processing of a face stimulus using MEG recordings [23]. We found that the amplitude of the ITG response had a similar pattern to the scalp N170. Indeed, the maximal amplitude of the ITG was evoked by fixations at the top of the face, regardless of its orientation. If the eye-processor hypothesis was valid, we would expect inverted faces, which cannot be processed holistically, to evoke larger N170 when fixations are at the eyes. In addition, we failed to find a cortical region that is particularly sensitive to fixations at the eyes, as source positions and extents were consistent among fixated face regions and face orientations. We note here however that our ability to discriminate face-sensitive from eventual eye-sensitive neural populations is limited by the spatial resolution inherent to any source reconstruction methodology (typically few cm). We can however reasonably argue that no eye-sensitive sources were detected in cortical areas distant from the ITG by at least few centimeters.

We further propose an alternate explanation for the amplitude of the N170 to eyes alone. Due to the large difference in stimulus size between eyes and whole faces, and consequently to the difference in the size of the cortical area activated by each stimulus, the observed differences may arise from source cancellation at the level of the N170 generators. Indeed, considering the geometry of the cortical ribbon, the amplitude of the electrical potential recorded over the scalp may decrease with source extent [24]. This is particularly the case when cortical activity recruits opposite walls of a sulcus. In order to asses this hypothesis in the ITG, we simulated EEG signals generated by cortical parcels with various locations in the ITG and spatial extents. We found cortical regions that show a regular decrease in EEG power with increasing spatial extent, within a specific range (delimited by extents A and B with 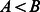, see fig. S3a). Importantly, spatial extent B corresponds to the size of the cortical region activated by whole faces (see fig. 6). This observation supports our hypothesis that source cancellation contributes to the observed larger N170 responses to eyes alone than to whole faces. Indeed, we verified that source cancellation potentially explains the difference in N170 amplitude between a region activated by a face and a smaller region (see fig. S3b). This hypothesis is consistent with the finding that faces produce similar N170 responses wether the eyes are present or not. We note here that the observed cancellation profiles are highly dependent on the location of the center of the parcel. More extensive study is needed to assess the consistency of the observed source cancellation and to characterize the cancellation profile in relation to the actual stimulus sizes reported in the previous studies. In addition, source cancellation is unlikely to explain weaker N170 responses to the nose and mouth than to whole faces. Additional factors, such as low contrast in the nose and mouth, must be taken into account when interpreting source cancellation results.

### Vertical asymmetry in neural face representations

Our study clearly demonstrates that by fixating the top of the face, the encoded visual information optimizes face representations in the brain. This result is in contradiction with those reported by McPartland et al. [10] who found that fixations on the nasion or on the mouth evoke N170 responses with similar amplitude. This latter study however suffers two limitations. Firstly, the actual gaze position within the faces was not monitored so neither the fixation position nor the number of fixations were strictly controlled. Secondly, the size of their stimuli (10.6°×8.1°) was smaller than the one used in the presented study (17°×23°); we can thus reasonably assume that the presented stimulus size allows a higher spatial resolution for studying the effects of changes in retinal projection on face-sensitive responses.

The most interesting result of our study is that the upper locations of a face stimulus elicit the highest N170 amplitude, either when stimulus is presented upright or inverted. One possible explanation for this observation is the distribution of photoreceptors within the fovea. Indeed, it is known that photoreceptors' distribution is denser in the upper half of the fovea [25], thus resulting in a more efficient sampling of the lower half of our visual field [26], . Many studies showed that objects present in the human lower visual field are processed more efficiently than objects in the higher visual field [25], [28]–[30]. Accordingly, a face stimulus will produce a higher cortical response when appearing in the lower visual field (i.e. gaze fixation on the top) than when appearing in the higher visual field (i.e. gaze fixation on the bottom). This interpretation further suggests that the effect of fixation position on face-sensitive responses are due to a general setup of our visual system. This, however, remains to be confirmed and our current research focuses on replicating our paradigm with different categories of visual stimuli.

### Retinotopy in face-responsive areas

As shown in fig. S1, when each of the fROI is foveated by the observer, visual field coverage of the face stimulus changes dramatically along both the vertical and the horizontal meridians. Fixations over lateral face regions, such as the left or right eyes and cheeks, produce asymmetrical distribution of retinal stimulation along the vertical meridian of the visual field. Consistent with the well-documented cross-hemisphere visual field maps of the peripheral signal, our results demonstrate a high sensitivity of ERP responses to lateral shifts of the observer's eye-gaze on the face image. We show that eye-fixations on lateral regions within faces elicited stronger responses at ipsilateral electrode sites than at contralateral sites, more specifically at the level of P100 responses. The effect of hemifield visual stimulation is also evidenced by the finding that stronger P100 and N170 responses were recorded over the right hemisphere when the fixated fROIs falls in the right than in the left part of the face stimulus, and over the left hemisphere when the fixated fROIs falls in the left part of the face.

Furthermore, fixations over upper and lower face regions, such as the nasion and the mouth, produce asymmetrical distribution of retinal stimulation along the horizontal meridian of the visual field. Both the P100 and the N170 responses show sensitivity to quantitative changes of retinal stimulation across lower and higher visual hemifields. When the nasion was foveated by observers, P100 responses over midline parieto-occipital sites were more positive for inverted than for upright faces. The reverse pattern was found when the mouth was foveated. This pattern of activity bears some similarity with previous studies showing VEP responses consistent with the retinotopic response map of V1 [31], [32]. N170 results show moderate effects of vertical asymmetry; N170 responses were the largest when the fixated fROI were located in the upper part of upright (i.e, nasion) and inverted face images (i.e., mouth). Moreover, the N170 was always more negative in response to inverted faces, whether inversion shifted the feature position downward or upward.

These findings provide support for a retinotopic organization of neural populations in low- but also high-order visual areas. While low-level visual areas exhibit an explicit retinotopic structure with small receptive fields, electrophysiological studies probing retinotopy of high-level visual areas provide contradictory results, mainly because of the high variability of receptive fields (20°–80°) in these areas [33]. When controlling precisely the lateral gaze shift with respect to stimuli, we observe a consistent horizontal eccentricity effect, suggesting that face responsive areas exhibit at least coarse retinotopic organization [34].

## Conclusion

In conclusion, our results suggest that the eyes *per se* do not play a special role for the structural encoding of human faces. More importantly, they raise the possibility that the effect of gaze location on face processing is based on a general retinotopic visual setting enhancing visual information located in the lower visual field rather than the neural representations processes specific to facial features. In order to corroborate this hypothesis, future work will focus on the gaze effect on processing of different stimulus categories, vertically oriented stimuli (e.g. house, animal faces) as well as laterally oriented stimuli (e.g. cars). In addition, we will also focus on top-down effects on this visual setting using emotional face stimuli. Although the present data were not acquired in a strictly naturalistic viewing condition, they firmly highlight the pitfalls of using highly unnatural scenes for the study of complex perceptual processes.

## Supporting Information

Appendix S1 **Localization of cortical sources using the maximum entropy on the mean (MEM) principle.**
(PDF)

Figure S1 **Spatial resolution of face images and visual field map coverage, along with visual field quadrants, when each of the seven fROIs is centred on the observer's line of sight.** When each of the fROI is foveated by the observer, vast changes occur both in terms of image spatial resolution and visual field coverage. The simulated face resolution displays were generated using the multiresolution pyramid method described in Perry and Geisler [35]. The algorithm keeps the high resolution region of the displayed image centred on the observer's line of sight while it mimics the precipitous fall-off in spatial resolution of the human visual system from the point of gaze [26], [36]. The distribution of the facial area, expressed in proportion of pixels, across the horizontal (left *vs.* right) and vertical (lower *vs.* upper) visual field quadrants differed markedly as a function of the location of eye-fixations.
(EPS)

Figure S2 **Heat maps for upright and inverted faces.** Values of the heat maps indicate the relative strength of P100 response as a function of viewpoint. No point on the heat map survived the 5% threshold with Bootstrap analysis.
(EPS)

Figure S3
**Source cancellation in the inferior temporal gyrus. a**) Simulation of the EEG recorded from the activity of different cortical parcels in the ITG as a function of their spatial extent. For each parcel and each spatial extent, the EEG global field power (GFP) was computed as the sum of variances recorded at all electrodes. We selected a group of regions (red line) showing source cancellation at the same spatial extent as the reconstructed regions (point B) with the MEM (see Fig. 6). The cancellation is indicated on the graph as the difference between the simulated GFP (red) and the interpolated GFP (magenta) based on the GFP produced smaller extents of the same source. We also observed parcels that show no source cancellation (blue line). The standard deviations for each group is depicted with a ribbon around the average. **b**) We assessed that source cancellation translates into differences in the amplitude of the N170 and that this difference is compatible with the reported N170 amplitudes evoked by faces and eyes alone. We simulated two N170 ERPs for each source that showed cancellation in a), one with the spatial extent A and one with the spatial extent B. We assume spatial extent A is the one recruited by the eyes alone, given that their size is smaller than whole faces. On PO7 and PO8, we observe average differences of 0.5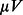 and 0.25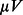, respectively.(EPS)
