# Supplementary material for: Optimal Eye-Gaze Fixation Position for Face-Related Neural Responses
Source: PLoS One. 2013 Jun 6;8(6):e60128. doi: 10.1371/journal.pone.0060128 (PMC3675086; doi:10.1371/journal.pone.0060128)
Supplement: Appendix S1 — Localization of cortical sources using the maximum entropy on the mean (MEM) principle. (PDF) [file pone.0060128.s001.pdf]

Appendix

The MEM is a probabilistic approach based on the entropy principle aimed at inverting the following data-generative model from a distributed source model with N dipolar sources q:

$$m(t) = G\mathbf{q}(t) + \eta(t) \quad (1)$$

with a solution of the form:

$$\mathbf{q} = E_p^*[\mathbf{q}] \quad (2)$$

$m(t)$ is the data at hand, G is the lead field matrix, $\eta(t)$ is a random noise. $E_p^*[\mathbf{q}]$ is the expectation of $\mathbf{q}$ under its posterior probability distribution $p^*(\mathbf{q})d(\mathbf{q})$ as obtained by the MEM. Throughout this section, the notation $dp(\mathbf{q})$ is used as a shortcut to $p(\mathbf{q})d\mathbf{q}$. The general idea of the MEM is detailed in two steps: the model priors and the optimal solution.

Priors

Te first step is to define a reference distribution of source intensities $d\mu(\mathbf{q})$ that encompasses all the prior information about our sources model. The originality of the current MEM framework is to describe the brain functional activity with K homogenous cortical parcels P_k. Each parcel gathers n_k dipoles and is characterized with a hidden state variable S_k that can be either 1 (active state) or 0 (inactive state). The probability of parcel k to be in the active state ($p(S_k = 1)$ is noted α_k. In practice, the reference probability distribution $d\mu(\mathbf{q}_k)$ of the sources within parcel k is a mixture between both states :

$$d\mu_k(\mathbf{q}_k) = ((1 - \alpha_k)\delta(\mathbf{q}_k) + \alpha_k \mathcal{N}(\nu_k, \Sigma_k)(\mathbf{q}_k))\, d\mathbf{q_k} \quad (3)$$

where δ is the Dirac distribution modeling the inactive state and $\mathcal{N}(\nu_k, \Sigma_k)$ is a k-dimension multivariate normal distribution of mean ν_k and covariance Σ_k modeling the active state of parcel k. As we assume that the cortical patches are independent, the general reference model $d\mu(\mathbf{q})$ can be written:

$$d\mu(\mathbf{q}) = \prod_{k=1}^{K} d\mu_k(\mathbf{q}_k) \quad (4)$$

The initial values for α_k are computed using the multivariate pre-localization formalism (MSP, [1]). The MSP scores, ranging between 0 and 1, measure the ability of each source to explain the data. We thus define:

$$\alpha_k = median(r_k) \quad (5)$$

where r_k is the set of MSP scores of the n_k sources within the parcel k. The probability α_k is subordinate to the definition of the parcels, which is achieved by running a region growth algorithm on the sources with highest MSP scores with a neighborhood constraint. In the present work, the size of the parcels is limited to a neighborhood order 4, thus each parcel typically gathers 50 dipoles.

Solution

The main computational problem is the estimation of the posterior probability distribution $dp^*(\mathbf{q})$ of source intensities. This distribution must explain the data under the constraint of maximizing the relative entropy $S_\mu(p)$. This entropy is a measure of the statistical distance between any probability distribution $dp(\mathbf{q})$ and a reference probability distribution $d\mu(\mathbf{q})$. $S_\mu(p)$ has a strictly negative or null value and is given by:

$$S_\mu(p) = -\int p(\mathbf{q}) \log \frac{p(\mathbf{q})}{\mu(\mathbf{q})} d\mathbf{q} \quad (6)$$

$S_\mu(p)$ can be interpreted as the effective amount of information from the data used to correct $d\mu(\mathbf{q})$. As $S_\mu(p)$ is either negative or null, maximizing the entropy amounts to minimizing the statistical distance between $dp(\mathbf{q})$ and $d\mu(\mathbf{q})$. The MEM principle minimizes the false assumptions about the data [2]. The numerical optimization problem for computing $dp^*(\mathbf{q})$ is detailed by Amblard et al. [3].

References

1. Mattout J, Plgrini-Issac M, Garnero L, Benali H (2005) Multivariate source prelocalization (msp): use of functionally informed basis functions for better conditioning the meg inverse problem. Neuroimage 26: 356-373.

2. Jaynes ET (1957) Information theory and statistical mechanics. Phys Rev 106.

3. Amblard C, Lapalme E, Lina J (2004) Biomagnetic source detection by maximum entropy and graphical models. IEEE Trans Biomed Eng 51.
